# The role of hyaluronan in renal cell carcinoma

**DOI:** 10.3389/fimmu.2023.1127828

**Published:** 2023-03-02

**Authors:** Chenchen Jin, Yunfeng Zong

**Affiliations:** ^1^ Zhejiang Academy of Science & Technology for Inspection & Quarantine, Hangzhou, Zhejiang, China; ^2^ The Cancer Hospital of the University of Chinese Academy of Sciences (Zhejiang Cancer Hospital), Institute of Basic Medicine and Cancer (IBMC), Chinese Academy of Sciences, Hangzhou, Zhejiang, China

**Keywords:** hyaluronan (HA), HA receptors, HA synthases, hyaluronidases, renal cell carcinoma (RCC), therapies

## Abstract

Renal cell carcinoma (RCC) is associated with high mortality rates worldwide and survival among RCC patients has not improved significantly in the past few years. A better understanding of the pathogenesis of RCC can enable the development of more effective therapeutic strategies against RCC. Hyaluronan (HA) is a glycosaminoglycan located in the extracellular matrix (ECM) that has several roles in biology, medicine, and physiological processes, such as tissue homeostasis and angiogenesis. Dysregulated HA and its receptors play important roles in fundamental cellular and molecular biology processes such as cell signaling, immune modulation, tumor progression and angiogenesis. There is emerging evidence that alterations in the production of HA regulate RCC development, thereby acting as important biomarkers as well as specific therapeutic targets. Therefore, targeting HA or combining it with other therapies are promising therapeutic strategies. In this Review, we summarize the available data on the role of abnormal regulation of HA and speculate on its potential as a therapeutic target against RCC.

## 1 Introduction

Kidney cancer represents around 3% of all cancer diagnoses and deaths worldwide, with a higher incidence being reported in developed nations (1, 2). Renal cell carcinoma (RCC) is the most common malignant tumor accounting for 80-85% of all kidney cancers (3). The three main histological subtypes of RCC are clear cell RCC (ccRCC, 70%), papillary RCC (pRCC, 10-15%) and chromophobe RCC (chRCC, 3-5%) (4, 5). Age, gender, race, geographic location (1, 6), obesity (7, 8), smoking (9, 10), and hypertension (11, 12) are associated with development of RCC, while lifestyle and dietary modifications may reduce risk of developing RCC (13). RCC is associated with high mortality rates because its poor sensitivity to therapies, and high recurrence risk after nephrectomy, providing a 60-70% 5−year survival rate (14). Metastasis is present in approximately 30% of RCC cases at initial diagnosis, which lead to poor clinical outcomes (15). Existing targeted immunotherapies and other therapeutic strategies against RCCs have limited efficacy, which has prompted interest in the development of alternative strategies (16).

Hyaluronan (HA) is a ubiquitous polyanionic glycosaminoglycan (GAG) found in the extracellular matrix (ECM) that also forms a pericellular coat surrounding cells. HA plays important roles in a variety of physiological functions, including cell motility and inflammation (17). Research has been conducted on the specific roles of HA in diseases such as cancer, rheumatoid arthritis and infectious diseases (18). Signal transduction and functions of HA depend on its molecular size. High molecular weight HA (HMW-HA; >500 kDa) promotes anti-inflammatory effects in most cases, whereas low molecular weight HA (LMW-HA; <120 kDa) acts as a pro-inflammatory “danger” signal that triggers local inflammation (19).

High levels of HA are associated with unfavorable prognosis in multiple cancers (20, 21). HA has recently emerged as a key player in nephrology and urology that plays a role in inflammation and ECM organization (22). However, there is no clear consensus on the importance of HA in RCC. Emerging evidence suggests that HA accumulation abnormally in RCC may contribute to aggressive malignancies and metastatic carcinomas, and may serve as an essential therapeutic target (23). Herein, we highlight the characteristics of HA and its main receptors in RCC, with specific focus on its abnormal regulation and potential as a therapeutic target.

## 2 HA biology and kidney

**Figure 1:**
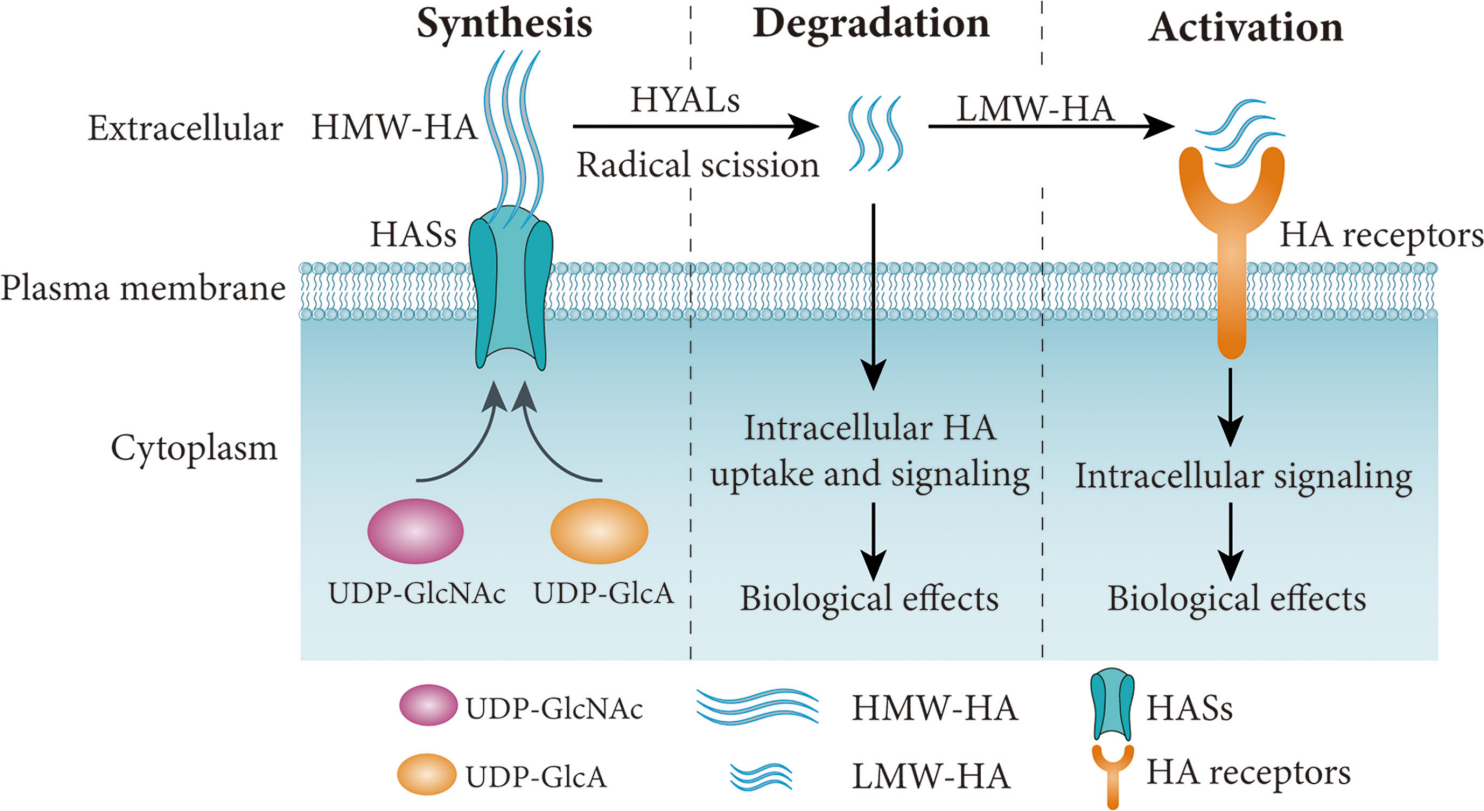
Simplified schematic diagram of HA synthesis, degradation and signaling pathways. HA is synthesized by HASs using UDP-GlcA and UDP-GlcNAc as substrates, and degraded into LMW-HA by HYALs or free radicals. The HA receptors are activated by LMW-HA and involved in various cellular functions. HA, hyaluronan; LMW-HA, low molecular weight HA; HASs, HA synthases; HYALs, hyaluronidases. Figure adapted from Ref (37, 38).

HA was independently identified by Meyer and Palmer in 1934, and was previously named from hyaloid and uronic acid (24). HA is a GAG synthesized by a wide range of living organisms. It consists of repeating disaccharide units of glucuronic acid (GlcA) and N-acetylglucosamine (GlcNAc) bound together (25). HA is well known for its water absorption abilities and its capacity to generate higher concentrations of gels (26). Eukaryotic cells use HA synthases (HAS1-3) to synthesize HA on their plasma membranes (**Table 1**, **Figure 1**). Among them, HAS1 is the least active enzyme, and requires a high concentration of UDP-GlcNAc to function (39), while HAS3 is the most active synthase. HAS1 and HAS2 synthesize HMW-HA, while HAS3 synthesizes LMW-HA (40). HAS2 is the primary HA synthase during development (41). In mammals, expression of HASs varies between normal and pathologic conditions based on tissue and cell types.

**Table 1:** HA synthases and hyaluronidases in humans.

Enzyme	Gene	Chromosome	Characteristics	Reference
HAS1	*HAS1*	19q13.3-19q13.4	The least active HASs; synthesize HMW-HA.	(27, 28)
HAS2	*HAS2*	8q24.12	More catalytically active; synthesize HMW-HA. The major HA synthase during development.	(27, 28)
HSA3	*HAS3*	16q22.1	The most active HASs; degrade HMW-HA into LMW-HA.	(27, 28)
HYAL1	*HYAL1*	3p21.3	pH optimum near 3.7.	(29, 30)
HYAL2	*HYAL2*	3p21.3	pH optimum of below 4; synthesize ~20 kDa fragments.	(30, 31)
HYAL3	*HYAL3*	3p21.3	pH optimum of below 4.	(30, 32)
HYAL4	*HYAL4*	7q31.3	Weak hyaluronidase activity.	(30, 32)
HYALP1	*HYALP1*	7q31.3	Pseudogene.	(30, 32)
PH20	*SPAM1*	7q31.3	Neutral pH; fertilization.	(30, 33)
TMEM2	*TMEM2*	9q21.13	Neutral pH; degrade HMW-HA into ~5 kDa fragments.	(34, 35)
CEMIP	*CEMIP*	15q25.1	Degrade HMW-HA into intermediate and LMW-HA.	(35, 36)

HAS, HA synthase; HYAL, hyaluronidase; TMEM2, transmembrane protein 2; CEMIP, cell migration-inducing protein.

HA undergoes rapid turnover in the ECM, with a third of the 15g mass in an average adult human undergoing turnover each day. An increase in HA levels may be associated with higher turnover, which may reflect the pathological conditions. HA undergoes turnover and catabolism after internalization by many tissues through receptor-mediated endocytosis. HMW-HA is degraded by hyaluronidases (HYALs) (32), reactive oxygen species (ROS) (42) or ultraviolet (UV) radiation (43) (**Figure 1**). HA is mostly excreted in the liver each day, while only 1-2% of HA is removed in the kidney (44–47). Excretion through the kidney is limited to LMW-HA (< 12 kDa) that can pass through the glomerular barrier. In mammals, the main members of the family of HYALs include HYAL1-4, PH20 and HYALP1 (**Table 1**). HYAL1 cleaves HA of different molecular weights (32), while HYAL2 degrades HMW-HA into approximately 20 kDa fragments (31). HYAL3 is widely expressed, while the expression of HYAL4 is mostly in the placenta and skeletal muscle. In humans, HYALP1 is expressed as a pseudogene, but its function is unclear. PH20 plays a role in fertilization and is almost exclusively expressed in the testes (33). However, PH20 is overexpressed in other malignant tissues, such as breast (48), prostate (49) and laryngeal (50) cancers. Proteins such as TMEM2 (transmembrane protein 2) and CEMIP (cell migration-inducing protein, also called HYBID or KIAA1199), are capable of depolymerizing HA (51, 52). HA degradation products such as LMW-HA and oligosaccharides (<10 kDa) activate signaling cascades that promoting inflammation and angiogenesis and are generally associated with pathological states (**Figure 1**), such as cancer (19).

HA is predominantly produced in the interstitium of the renal papilla (medulla) in normal kidney, while its production in the renal cortex is very low accounting for 1-3% of the production in the medulla (53–57). The gene expression levels are HAS2 > HAS1 > HAS3 (58, 59). It is important to note that RCC mostly originates from the renal cortex. Extracellular HA is degraded by HYAL2 in all kidney regions, whereas intracellular HA is degraded by HYAL1 (60). Normal kidneys have low production of HA, with increased production of HA in the renal interstitium being linked to several renal diseases, such as acute kidney injury (61), chronic kidney diseases (62), allograft rejection (63), diabetic nephropathy (64), obstructive uropathy (65), IgA nephropathy (66), and kidney stones (67). It has been proposed that altered production of HA in papillary interstitial tissues regulates renal water handling through its effects on the matrix’s physiochemical properties and interstitial hydrostatic pressure (55, 68, 69). Ito et al. (70) demonstrated that CD44 in renal proximal tubular epithelial cells (PTCs) modulates HA-mediated regulation of cell function through TGF-β mediated mechanisms. Van den Berg et al. (71) found that glomerular endothelial HA contributes to glomerular structure and function, but whose production is lost in diabetic nephropathy. In addition, immune mediators may induce cortical fibroblasts to produce more HA (54, 72), suggesting that inflammation causes accumulation of HA in the cortex. There is need for further studies to identify the exact nature of the cells responsible for HA synthesis or the factors that contribute to its increased production in kidney diseases like RCC.

## 3 TME, ECM and immunity

Cancer is a complex systemic disease. The tumor microenvironment (TME) is composed of tumor cells and adjacent noncancer components, such as immune cells, fibroblasts, ECM and many others (73). Constant interactions between tumor cells and other components constitute a highly complex, dynamic and heterogenous network of the TME that supports tumor growth and invasion (74). RCC is associated with high infiltration of several immune cells, making it one of the most immunoreactive tumors (75–78). It also consists of various myofibroblasts and endothelial cells (79). Targeting cancer cells in the TME has become an appealing strategy for treating RCC (80, 81). Better understanding of the RCC may lead to the identification of specific therapeutic targets in the microenvironment, which can be used to improve the prognosis of patients.

The ECM is highly dysregulated in cancer, and may play pro-tumorigenic or anti-tumorigenic roles. During cancer progression, ECM recognizes various cell surface receptors and initiates signaling pathways that promote tumor growth (82). Unlike the ECM in healthy kidneys, RCC ECM represent is composed of a complex network of components such as GAGs, collagen, fibronectin, tenascin C, and laminins (83, 84). GAGs are regulated by altered metabolic pathways in RCC, which are associated with tumor aggressiveness and recurrence (85–87). HA is a widely produced GAG of the ECM that can have tumor promoting or tumor suppressing roles. Meanwhile, HA is mostly produced in tumor cells as well as cancer-associated fibroblasts (CAFs) in the TME (88), with the level of production varying according to the stage of the tumor. Many pro-tumorigenic effects are attributed to HA fragments (89). Under steady-state conditions, HMW-HA (>500 kDa) is the dominant HA size in most tissues and inhibits tumor progression, while LMW-HA (<120 kDa) may regulate tumor growth, invasion and metastasis through HA receptors in TME, such as CD44 and RHAMM (19, 88). Size-specific HA signaling may be related to unique conformational changes in the external receptor•HA complexes (90).

In some tumors, tumoral HA and its degradation products induce tumor angiogenesis and activate both innate and adaptive immune responses (91–93), but this association has not been studied in RCC. Tumoral HA is known to recruit tumor-associated macrophages to promote tumor neovascularization (94). LMW-HA can induce dendritic cells (DCs) activation and maturation, release proangiogenic cytokines and modulate proangiogenic properties in TME (93). Furthermore, HA has frequently been implicated in T cell trafficking and induction of cell death in activated T cells through CD44 (95, 96). Regulatory T cells (Tregs) are potent immunosuppressive cells that promote tumor angiogenesis (93) with HA binding populations being functionally more suppressive (91). In addition, interaction between natural killer (NK) cell receptor and HA on tumor cells possible to augment NK cell cytotoxicity (97). Thus, it is not surprising that HA plays significant roles in the regulation of tumor immunosuppression.

## 4 HA in RCC

Increased production of HA in tumor parenchyma, TME or serum is associated with tumor growth and poor outcome in cancer patients, including RCC (23), breast cancer (98), head and neck squamous cell carcinoma (99), lymphomas (100), gliomas (101), melanomas (102), lung carcinomas (103), hepatocellular carcinoma (104), and other cancers. Kaul et al. (22) reported several kidney diseases, including RCC, which are associated with changes in production of HA. Jokelainen et al. (23) revealed that 39.6% of RCC samples were HA positive. Furthermore, high cellular HA was associated with higher tumor grades and was a marker of poor prognosis in RCC patients. Thus, tumoral HA may play a role in the progression of the cancer and may act as a prognostic factor for RCC.

Zoltan-Jones et al. (105) reported that β-catenin regulated HA production in Madin-Darby canine kidney (MDCK) cells and could lead to epithelial-mesenchymal transition (EMT). Rilla et al. (106) found that induction of HAS3 expression in MDCK cells may be related to premalignant phenotypes. Moran et al. (84) reported that HAS1 regulated the migration of renal carcinoma *in vitro* and found no distant metastasis in mice after implanting HAS1-deficent cells. Recent evidence demonstrates that microRNA-125a may play a role in the progression of RCC through interaction with HAS1 (107), suggesting that the tumor promoting properties of HA can be explained by another mechanism.

Chi et al. (108) used Q-PCR to compare gene expression between tumor tissues and adjacent normal tissue and found that HAS1 levels were increased in ccRCC, pRCC and chRCC tissues. The expression of HYAL4 in ccRCC and pRCC was higher than in oncocytomas, while the expression of HYAL1 was lower in ccRCC than in normal kidney. There was no difference in expression between normal and tumor tissues among other members of the HA family including HAS2, HAS3, HYAL2, HYAL3, PH20, HYALP1 and CD44v. Cai et al. (109) found that the expression of HAS1-3 mRNA in human ccRCC was higher than that in adjacent normal renal samples. However, only the HAS3 protein expression was higher in ccRCC tissues at the protein level. Immunohistochemical staining showed that weak HA staining in human ccRCC tissues compared with normal adjacent samples. Similarly, Ucakturk et al. (87) used UPLC-MS analyses to show that no difference was found in HA production between RCC and normal renal samples. It is putative that HASs transcription or protein expression levels in human RCC might not reflect HA levels. Taken together, reports on the expression patterns of the HA family members in RCC are inconsistent and may be due to different experimental conditions. Also, additional mechanisms could be involved. Thus, the exact role of HA in RCC is uncertain, and further studies that are more sensitive and specific are required.

Kusmartsev et al. (110) observed an increase in HYAL2^+^PD-L1^+^ myeloid-derived suppressor cells (MDSCs) in ccRCC tumor tissue and peripheral blood. Furthermore, stroma-associated PD-L1^+^ myeloid cells showed significant production of HA. HYAL2^+^ myeloid cells indicate the occurrence of HMW-HA degradation into LMW-HA, suggesting that the relationship between myeloid cells and HA may be involved in the promotion of cancer-related inflammation and immune functions. Similarly, Dominguez-Gutierrez et al. (111) found that LMW-HA was accumulated by HYAL2^+^ tumor associated myeloid cells in human bladder cancer and associated with elevated production of tumor angiogenic factors. Unfortunately, it is still unclear how HA-immune interactions occur in RCC.

## 5 HA receptors in RCC

HA also interacts with specific proteins (**Table 2**) called hyaladherins (129) such as TSG-6 (130), and various cell receptors, including CD44 (131), receptor of HA-mediated motility (RHAMM) (113), layilin (132), lymphatic vessel endothelial receptor 1 (LYVE1) (118), intracellular adhesion molecule 1 (ICAM1) (117), toll like receptors (TLRs) (114), and hyaluronic acid receptor for endocytosis (HARE or Stabilin-2) (120). The receptors are activated by LMW-HA and are involved in various cellular functions including tumor metastasis and lymphocyte activation. For example, LMW-HA regulates breast cancer progression through CD44 and TLRs signaling (133). Only a few studies have investigated the cellular mechanisms underlying the role of HA receptors in RCC pathogenesis.

**Table 2:** The roles of major hyaladherins in cancer.

Hyaladherin	Gene	Main functions	Reference
CD44	*CD44*	Carcinogenesis and signaling regulator.	(112)
RHAMM	*RHAMM*	Tumor cell migration and oncogenesis.	(113)
TLR2/4	*TLR2, TLR4*	Tumor growth and lymph node metastasis.	(114–116)
ICAM1	*ICAM1*	Cell adhesion, tumor progression.	(117)
LYVE1	*LYVE1*	Tumor lymphangiogenesis.	(118, 119)
HARE	*HARE*	Tumor metastasis.	(120)
Layilin	*LAYN*	Negative regulator.	(121)
TSG-6	*TSG6*	Inflammation and tumor metastasis.	(122, 123)
SHAP	*ITIH1*	Tumor metastasis.	(124, 125)
HABP1	*HABP1*	Tumor metastasis and invasion.	(126)
Brevican	*BCAN*	Tumor invasion.	(127, 128)
Neurocan	*NCAN*	Tumor invasion.	(127, 128)
Versican	*VCAN*	Tumor growth and angiogenesis.	(29)

CD44, cluster of differentiation 44; RHAMM, receptor of HA-mediated motility; TLR, toll like receptor; ICAM1, intracellular adhesion molecule 1; LYVE1, lymphatic vessel endothelial receptor 1; HARE, hyaluronic acid receptor for endocytosis; TSG-6, tumor necrosis factor-(TNF) stimulated gene-6; SHAP, serum-derived hyaluronan associated protein; HABP1, hyaluronan-binding protein 1.

CD44 proteins are primary HA receptors that promote invasion and metastasis of cancer cells by modulating intracellular signaling through its interaction with RHAMM (131, 134). RHAMM regulates cell proliferation and transformation and is overexpressed in most cancers (113), the expression of RHAMM is an independent prognostic factor for RCC (135). Chi et al. (108) found that RHAMM was significantly higher in ccRCC, chRCC and pRCC than in normal kidneys. Expression of CD44s and RHAMM was also higher in ccRCC and pRCC than in oncocytomas. These findings indicate that RHAMM and CD44s expression levels in RCC tissues are potential predictors of metastasis. Furthermore, HA and proteoglycan link protein 3 (HAPLN3) is overexpressed and may promote tumor progression in ccRCC through immune cells infiltration (136).

Layilin is a HA receptor homologous to C-type lectin that has been reported to regulate cell adhesion and migration through binding to cytoskeletal proteins such as merlin and talin (137). The prognostic value of layilin in hepatocellular carcinoma was reported by Zheng et al. (121), who concluded that layilin is an unfavorable risk factor since it suppresses the functions of the CD8^+^ T cells in TME. In contrast, Mahuron et al. (138) reported that layilin enhanced the cytotoxic potential of melanoma. Research on mice has shown that layilin is expressed in various organs, including kidney or normal rat kidney cell line (137). Adachi et al. reported that layilin silencing prevented EMT in human ccRCC *in vitro* (139). These results implied that the exact function of layilin remains unclear.

In the renal papillae, HARE is localized to the endothelial cells that internalize circulating HA (140). Tissues with the highest expression of HARE are the most common targets of metastatic cancer (120). LYVE-1 is another HA-binding receptor that is found in the lymphatic vascular endothelial cells and renal tubular epithelium cells (141, 142). LYVE-1 has been used to map lymphatic vessels within and around tumor tissues to determine patient survival (143). Unfortunately, there are no reports on the roles of LYVE-1 and other hyaladherins in RCC.

## 6 Potential therapeutic applications of targeting HA in RCC

**Figure 2:**
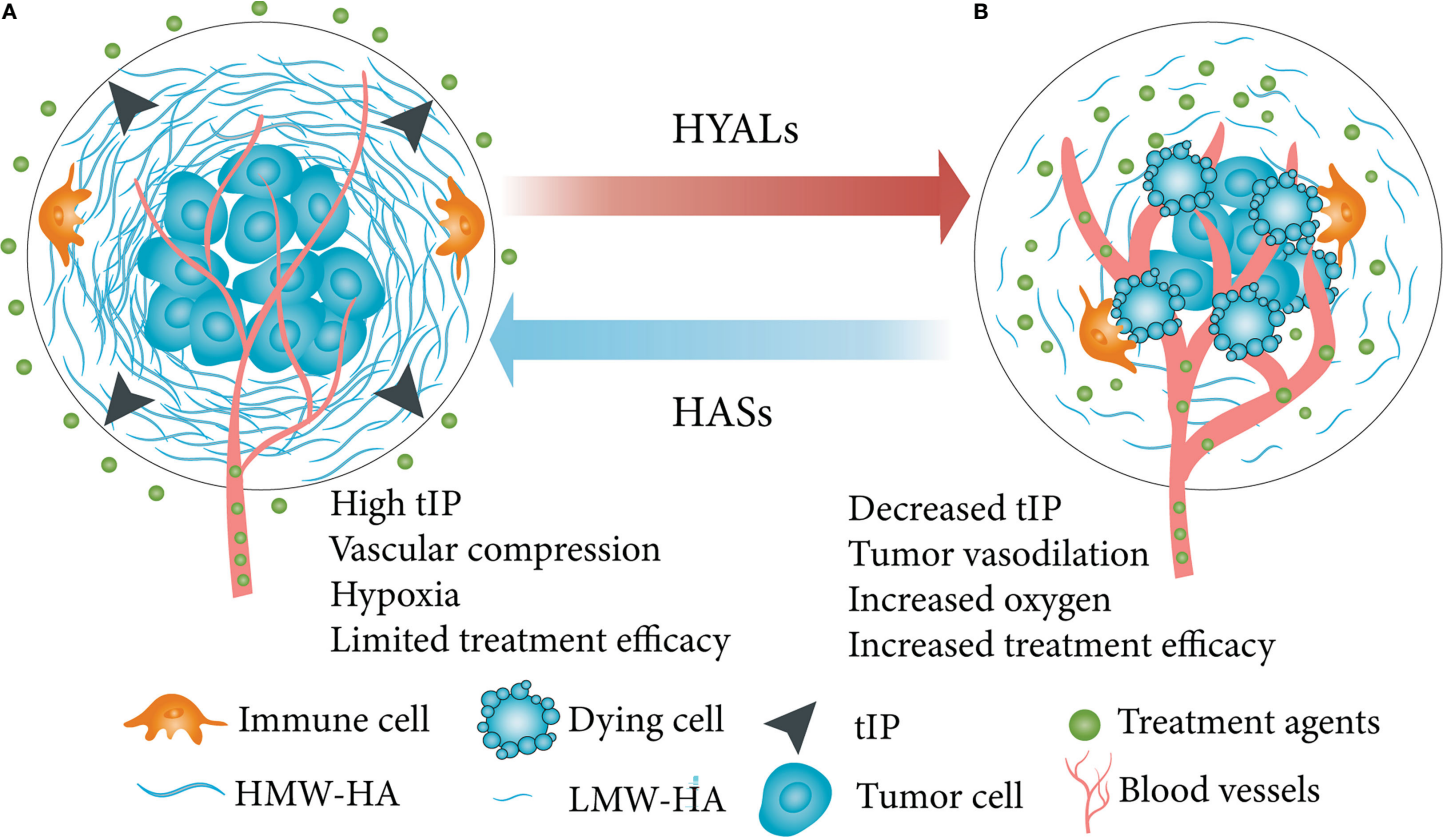
Simplified schematic diagram of HA depletion in a HA-enriched tumor phenotype. **(A)** HA swelled the TME, increased tIP, compressed tumor vasculature, caused tumor hypoxia and other effects, then limited the effectiveness of therapeutic molecules. **(B)** HA depletion in tumor led to vascular permeability, improved drug penetration, inhibited tumor growth and improved therapeutic efficacy. HA, hyaluronan; TME, tumor microenvironment; tIP, tumor interstitial pressure; HYALs, hyaluronidases. Figure adapted from Ref (145).

HA deposition persists in the TME contributes to pathophysiology through induction of high tumor interstitial pressure (tIP) and compression of tumor vessels, which results in tumor hypoxia (144, 145). The ability of HA to cover specific epitopes with enriched pericellular matrix suggests that HA could act as an immune regulator in human diseases, allowing affected cells to evade cellular immune attack (20, 146, 147). For instance, McBride et al. (148) reported that the HA pericellular matrix inhibits the formation of synapses by immune cells and killing malignant cells *in vitro*. In addition to formation of pericellular coats *in vitro*, HA can also form cables that may facilitate communication between cells (149). Breaching the HA barrier from the tumor leads to vascular permeability and improved drug delivery, monoclonal antibody (mAb), cytotoxic chemotherapy or immune cell therapeutic efficacy (**Figure 2**).

Research on HA signaling suggests that targeting HA and other members of the HA family could be used to treat cancer (150). For instance, inhibition of HAS1 induced apoptosis in bladder cancer *in vitro*, thus inhibiting tumor growth and angiogenesis (151). 4-methylumbelliferone (4-MU) is the best characterized chemical inhibitor of HA that inhibits HA synthesis by downregulating HAS2 and HAS3. It has been reported that 4-MU has potent anticancer effects in various tumors, including pancreatic cancer (152), breast cancer (153), esophageal cancer (154), skin cancer (155), bone cancer (156), leukemia (157), ovarian cancer (158), prostate cancer (159) and liver cancer (160). Additionally, HAS2 and HAS3 knockdown mimic the effects of 4-MU in esophageal squamous carcinoma cells (161).

Chemical compounds, such as sulfated HA (sHA), that have the ability to target HA degradation have been shown to inhibit the growth of prostate cancer cells and induce apoptosis (162). Similarly, sHA inhibits proliferation, motility, and invasion of breast cancer models (163). The depletion of HA in TME using HYALs is also being investigated as a potential cancer therapeutic strategy. PEGPH20 is human recombinant HYAL that depletes stromal HA in several animal models, and may induce reduction in tIP, increased penetration of tumors by drug as well as immune cells and inhibit the growth of tumor cells (164, 165). A variety of clinical trials are being conducted for various cancers using a combination of HYALs, chemo or radiotherapy (clinicaltrials.gov). However, whether depletion of HA could be applied to treating RCC is still unknown.

Since CD44 is a key receptor for HA, it has been targeted in different therapeutic strategies against cancer, such as vaccines, anti-CD44 antibodies, and nanoparticles that deliver CD44 siRNA (166). However, several phase I trials investigating CD44-targeted therapies showed limited clinical success in treating cancer, and the occurrence of severe side effects led to the termination of the project (167). Hence, targeting CD44 as a cancer therapeutic target requires careful evaluation. Hirose et al. (168) suggested that inhibition of HARE could be a potential strategy for preventing metastasis of melanoma to the lung in mice. Studies by Gahan et al. and Benitez et al. showed that combination of 4-MU and sorafenib inhibits the growth and motility of RCC cells by targeting RHAMM expression (169, 170), offering a potential pathway for therapeutic intervention in RCC. In combination with 4-MU, sorafenib also targets HAS3 and inhibits the growth of microvessels in RCC (171). Additionally, HA is an attractive candidate for conjugation to antitumor drugs or for use in nanoparticles (172–174). Chemotherapy drugs can be effectively delivered through HA nanomaterials. This may possibly increase the efficacy of chemotherapeutics or other therapies in tumors.

## 7 Conclusions and future perspectives

HA signaling pathway (HASs, HYALs, and HA receptors) is important in promoting tumor growth, metastasis, angiogenesis, and immune response. Therefore, potential therapeutic methods that can be developed include suppression of HA synthesis, clearance of the existing HA, and conjunction of HYALs and HA receptors with chemotherapy. Further studies are needed to identify the molecular mechanisms underlying the relationship between HA production and the development of cancers like RCC. There is also need to comprehensively profile the genes, proteins and metabolites involved in HA metabolism in RCC, since the whole signaling cascade is crucial to maintaining pro-cancer conditions. If these emerging strategies are clinically effective against RCC, then they could be used as adjuvant therapy in early disease to provide RCC patients with new options for the future treatment.

## Author contributions

YZ led and wrote the first manuscript. CJ contributed to writing and editing of final manuscript. All authors contributed to the article and approved the submitted version.
